# Determinants of diabetic nephropathy in Ayder Referral Hospital, Northern Ethiopia: A case-control study

**DOI:** 10.1371/journal.pone.0173566

**Published:** 2017-04-12

**Authors:** Solomon Hintsa, Lamessa Dube, Mebrahtu Abay, Teklit Angesom, Abdulhalik Workicho

**Affiliations:** 1 Department of Public Health College of Health Sciences, Aksum University ksum, Ethiopia; 2 Department of Epidemiology, College of Health Sciences, Jimma University Jimma, Ethiopia; University of Colorado Denver School of Medicine, UNITED STATES

## Abstract

**Background:**

Diabetic nephropathy is the most serious complication of diabetes which leads to end-stage renal failure and other complication of diabetes mellitus. Determinants of Diabetic nephropathy are not consistent in different studies and associated factors to chronic complications of diabetes are not specific and there are limited studies specific to diabetic nephropathy. Thus, the aim of this study is to identify determinants of diabetic nephropathy in Ayder Referral Hospital, Northern Ethiopia.

**Methods:**

A case-control study was conducted from February 14 to May 8 2016. Diabetic patients who developed nephropathy in the last two years were the cases and diabetic patients free of nephropathy were controls. Cases and controls were identified detailed review of the chronic care follow up chart. Then simple random sampling was used to select sample of 420 (with control to case ratio of 4:1) resulting in 84 cases and 336 controls. Record review and interviewer administered questionnaire were used to collect data. Data was coded and entered in to Epi-Data version 3.1 and then exported to STATA 12 for analysis. Variables with P-values< 0.25 in Bivariate logistic regression were selected for multiple logistic regressions to determine independent determinants of diabetic nephropathy. OR was calculated with 95% CI to show strength of association.

**Result:**

The mean age (±Standard deviation) for the cases and the controls were 52(SD: ±1.34) and 42.4(SD: ±0.8) respectively. In multiple logistic regressions age of patient (AOR: 1.037 95%CI: 1.01–1.064), duration of diabetes after diagnosis (AOR for one year increase: 1.09 95%CI: 1.036–1.15), not-adhered to blood glucose measurement at home (AOR: 6.81 95%CI: 1.15–40.24), having Systolic Hypertension (AOR;2.13 (1.002–4.51), poor glycemic control (AOR;2.71 95%CI: (1.49–4.95), being overweight(AOR;2.7(1.47–4.96) were the independent predictors of diabetic nephropathy.

**Conclusion:**

In the light of these findings, targeted interventions should be designed at the follow up clinic to address the risk of developing diabetic nephropathy among the risk groups.

## Background

Diabetes mellitus (DM) is chronic non-communicable disease characterized by hyperglycemia and it is globally an emerging public health problem. This disease is increasing morbidity and mortality because of chronic complications in the world. The chronic complications are cardiac disease, neuropathy, retinopathy, and nephropathy and foot ulcer. Diabetic nephropathy is the serious complication of DM which leads to end-stage renal failure and other complication of diabetes mellitus[1–5].

Estimates for 2013 by the International Diabetes Federation (IDF) in the world indicated that the number of adult patients with DM will expand from 381.8 million to 591.9 million which is by an increment of 55% in 2035; but, in Sub Saharan Africa, the projection is with an increment of 109.6% [6]. According to the WHO projection report of 2000, the number of diabetic patients in Ethiopia will be more than double by the year 2030[7].

Diabetic nephropathy (DN) is a clinical syndrome characterized by the occurrence of persistent microalbuminuria in concomitance with insulin or non-insulin dependent diabetes. It is diagnosed by persistent increment of albumin or protein in urine when there is no any other known renal disease. The end product of the consequence of DN end stage renal disease (ESRD) leading to total kidney failure. ESRD then leads to the need for dialysis or kidney transplantation[8–11]. DN is proposed to be more common among African patients those in the developed world. The prevalence of proteinuria in diabetes patients ranges all over the world, which most done in DM clinics, the prevalence of diabetic nephropathy varies from 2% to 53.1% [1,10,12–17] in the world and 5.3% to 53.1% in Africa[6,18]. Ninety five percent of DM patients in Africa develop proteinuria within 10 years of diagnosis, 35% develop end stage renal disease within 3 years of diagnosis and 18% die of diabetic nephropathy after 20 years of diagnosis [18]. Prevalence of diabetic nephropathy in Ethiopia, from the studies of diabetic complications, is 15.7% to 29.5%[7,19,20]. Fifteen percent of DM patients also develop diabetic nephropathy in Black lion referral hospital. From the estimations above, if the prevalence of diabetic nephropathy continue like this, there will be 270,000 cases of diabetic nephropathy in Ethiopia [21].

The most common risk factors related to complications of diabetes are socio demographic characteristics like age and sex, body mass index (BMI), hypertension, poor glycemic control, specific medication, negative attitude towards diabetes, poor treatment adherence, unhealthy diet, type of diabetes(type II DM), cholesterol level, smoking, alcohol drinking, fasting blood sugar and duration of diabetes[1,17,19,20,22] But the above factors are different across the studies.

Diabetes patients visiting health institutions in Ethiopia are increasing nowadays. Studies related to diabetic nephropathy scarce in Ethiopia and in Africa. Most of the studies related to predictors or determinants are concluded with all complications as the same variable whether is any complication or not. Most of the studies in Ethiopia have no consistent associated factors to diabetic nephropathy. There are limited studies that determine factors associated diabetic nephropathy in Mekelle, Ethiopia. This study aimed to identify determinants of diabetic nephropathy in Ayder referral Hospital, Northern Ethiopia.

## Methods

### Study design and setting

A case-control study was conducted in Ayder referral Hospital from February 14 to May8, 2016. Ayder referral Hospital is found in Mekelle city 783 Km north of Addis Ababa. It is the largest Hospital in the region which has more than 500 beds. The hospital serves patients from all parts of the region (which comprises about 5 million people) and other neighborhood regions such as Afar and Amhara regional states. There are 50 specialists, 21 general practitioners, 497 nurses, 28 midwifes, 75 laboratories and 73pharmacies. This Hospital gives service for 100,000 peoples per year (*http://www.mu.edu.et/chs/index.php/facilities/ayder-referral-hospital-staff-profile*). In the Hospital, there are around 2000 diabetic patients who follow treatment. Diabetic patients without complication follow their treatment every three month but, those who have complications are being appointed as per need by the physician to follow the complication.

### Study population

Diabetic patients who developed nephropathy within two years were defined to be casesof the study. Those diabetic nephropathies were who have history of diabetic nephropathy from the patient index card diagnosed by physicians. Diabetic patients free of nephropathy without any restriction time were controls of the study.

Sample size was determined by using Epi Info statistical software to determine two population proportion from the factors reviewed which gives maximum value [23] by using 95% CI, power 80%, control to case ratio 4 (because it is rare case), OR = 3 which is the ratio of odds of systolic hypertension among patients with proteinuria to odds of systolic hypertension among patients free of proteinuria`, probability of exposure to systolic hypertension among patients free of proteinuria = 7.41% and probability of exposure to systolic hypertension among patients with proteinuria as 19.36%.The calculated sample size was 400 (80 cases and 320 controls). By adding 5% non-response rate, the total sample size was 420(84 cases and 336 controls). Record review was conducted to identify diabetic nephropathy and free of nephropathy. Following this, cases were selected from diabetic nephropathy patients and controls from free of nephropathy diabetic patients by simple random sampling.

### Data collection process

Record review of diabetic patients was conducted to identify cases and controls from record by using checklist. Cases and controls were recorded by identification number. After cases and controls were differentiated, data was collected from record review/patient index card and interview of the study participants. Checklist and structured questionnaire were used for data collection. The questionnaire was prepared in English, translated to Tigrigna language then back translated to English. The questionnaire was pretested in 5% of the sample size in Mekelle Hospital. Data was collected by 3 trained health professionals (nurse and health officer).

The questionnaire which has three parts was developed by the principal investigator. The first part is socio demographic factors. The second part is behavioral factors like adherence, knowledge on and attitude towards diabetic care, smoking status, drinking alcohol and traditional herbs. Knowledge part has 10 items and each was evaluated as 0 or 1. Attitude part has 10 items and was graded in a Likert scale ranging from 1 to 5 and the values were reversed for negative statements. The parts for attitude and knowledge were tested for reliability and the test result showed Cronbach’s alpha of 0.61 which was taken to be satisfactory. The third part is medical history which was taken from record like duration of diabetes after diagnosis, type of diabetes, presence of diabetic nephropathy, presence of complications other than nephropathy, weight, height, blood pressure, and fasting blood sugar near to diagnosis of diabetic nephropathy for cases and near to data collection for free of diabetic nephropathy to related whether the factors comes first. As confirmed during the data collection professionals in the diabetic Clinic measures weight, height and blood pressure as follows. Weight was measured in light closing and without shoes in kilograms (kg) using calibrated UNICEF Seca digital weighing scale (Germany) at a precision of 0.1kg as per recommended but not repeated measurements[24,25]. Height was measured using Stadiometer (Seca, Germany) in centimeter (cm) in erect position that the back of the head, shoulder blades, buttocks, and heels make contact with the backboard at a precision of 0.1cm with shoes removed as per recommended but not repeated measurements [24,25]. Blood pressure was measured using a mercury sphygmomanometer with a cuff deflation rate of 2mmHg. Two measurements from left arm 5 minutes apart in sitting position was averaged to be recorded[24,25].

### Data processing and analysis

Collected data was edited, coded and entered in to Epi-Data version 3.1 and then exported to STATA 12 for analysis. Checking data code and data cleaning was done before analysis. Frequencies and cross tabulations was used to summarize descriptive statistics.

The association between diabetic nephropathy and covariates was assessed first by Bivariable logistic regression. Variables with P-value <0.25 were taken to multiple Logistic regression. Backward likelihood ratio with 0.1 probability removal was used to develop the model. OR was estimated with 95% CI to show strength of association and P-value < 0.05 was used to declare statistical significance. Goodness of fit of the final model was checked using Hosmer Lemeshow test of goodness of fit considering good fit at P-value> = 0.05, omnibus likelihood test <0.05 and model classification of accuracy was checked. Multicollinearity was assessed by variance inflation factor (VIF) using standard errors of the Beta coefficients at >2.

### Ethical consideration

The study has obtained ethical approval from Jimma University Institutional Review Board before its commencement. The aim of the study was explained and informed written consent was obtained from the study participants. Permission letter was obtained from Tigray Regional Health Bureau, and from Ayder referral Hospital medical director. The minimal anonymized data set is attached with the manuscript (S1 Data).

## Results

From a sample of 420 (84 cases and 336 controls), 409(84 cases and 325 controls) participated in this research.

The mean age (±Standard deviation) for the cases and the controls were 52(SD: ±1.34) and 42.4(SD: ±0.8) respectively. Forty-nine (58.33%) of cases and 178(54.77%) of controls were male participants. Majority of the participants, 77(91.67%) of cases and 233(71.69%) of controls, were from urban residence. In addition, the majority, 83(98.81%) cases and 310(95.38%) of controls belongs to Tigrian ethnicity (Table 1).

**Table 1:** Socio demographic characteristics of diabetic patients who follow at Ayder referral Hospital, Ethiopia, 2016.

**Variable**	**Category**	**Cases**	**Controls**
		Number (%)	Number (%)
Sex	Male	49(58.33)	178(54.77)
	Female	35(41.67)	147(45.23)
Residence	Urban	77(91.67)	233(71.69)
	Rural	7(8.33)	92(28.31)
Ethnicity	Tigrian	83(98.81)	310(95.38)
	Amhara	0(0)	10(3.08)
	Afar	1(1.19)	3(0.92)
	Others	0(0)	2(0.62)
Religion	Orthodox	78(92.86)	300(92.31)
	Muslim	6(7.14)	25(7.69)
Educational status	Illiterate	12(14.28)	64(19.69)
	Literate	72(85.72)	261(80.31)
Occupational status	Farmer	11(13.10)	72(22.16)
	Government employer	33(39.29)	77(23.69)
	Self and private business	32(39.09)	83(25.54)
	Non-employed and others	8(9.52)	93(28.51)
Marital status	Single	10(11.91)	84(25.85)
	Married	68(80.95)	219(67.38)
	Divorced	2(2.38)	4(1.23)
	Widowed	4(4.76)	18(5.54)
Income	= <1500 ETB	32(38.10)	194(59.69)
	>1500 ETB	52(61.90)	131(40.31)

In bivariate logistic regression age, residence, marital status, occupational status, income level, adherence to meal, adherence to exercise, adherence to blood glucose measurement at home, alcohol consumption, duration of DM, type of DM, complications other than diabetic nephropathy, systolic hypertension, diastolic hypertension, glycemic control and BMI were P-value <0.25 that enter to multiple logistic regression.

In multiple logistic regression age, adherence to blood glucose measurement at home, duration after diagnosis of diabetes, systolic hypertension, glycemic control and BMI were significantly associated with the development of diabetic nephropathy. Age of patient (AOR: 1.037 95%CI: 1.01–1.064), duration of diabetes after diagnosis (AOR for one year increase: 1.09 95%CI: 1.036–1.15), not-adhered to blood glucose measurement at home (AOR: 6.81 95%CI: 1.15–40.24), having Systolic Hypertension (AOR;2.13 (1.002–4.51), poor glycemic control (AOR;2.71 95%CI: (1.49–4.95), being overweight (AOR;2.7(1.47–4.96) were the independent predictors of diabetic nephropathy (Tables 2 and 3).

**Table 2:** Factors associated with diabetic nephropathy in Ayder referral Hospital, Ethiopia, 2016.

Variables	Category	Cases (n = 84)	Controls (n = 325)	COR (95% CI)	P-value	AOR (95% CI)
**Age**		84	335	1.05(1.03–1.07)	<0.001	1.037(1.01–1.064)*
**Sex**	Male	49(58.33)	178(54.77)	1.16(0.71–1.88)	0.558	
	Female	35(41.67)	147(45.23)	1		
**Residence**	Urban	77(91.67)	233(71.69)	4.34(1.93–9.77)	<0.001	
	Rural	7(8.33)	92(28.31)	1		
**Educational status**	Illiterate	12(14.28)	64(19.69)	1.47(0.75–2.87)	0.258	
	Literate	72(85.72)	261(80.31)	1		
**Occupational status**	Farmer	11(13.10)	72(22.15)	1		1
	Government employee	33(39.29)	77(23.69)	2.81(1.32–5.96)	0.007	1.41(0.57–3.49)
	Private business	32(39.09)	83(25.54)	2.52(1.19–5.37)	0.016	1.122(0.43–2.92)
	Non-employed#	8(9.52)	93(28.51)	0.56(0.22–1.47)	0.242	0.36(0.11–1.83)
**Marital status**	Single	10(11.91)	84(25.85)	1		
	Married	68(80.95)	219(67.38)	2.61(1.28–5.3)	0.008	
	Others$	6(7.14)	22(6.77)	2.29(0.75–6.99)	0.145	
**Income**	= <1500 ETB	32(38.10)	194(59.69)	1		
	>1500 ETB	52(61.90)	131(40.31)	2.41(1.47–3.94)	<0.001	
**Medication Adherence**	Adhered	80(95.24)	316(97.23)	1		
	Not adhered	4(4.76)	9(2.77)	1.76(0.53–5.85)	0.359	
**Adherence to diet**	Adhered	48(57.14)	130(40.00)	2(1.23–3.25)	0.005	1.71(0.13–16.67)
	Not adhered	36(42.86)	195(60.00)	1		1
**Adherenceto exercise**	Adhered	35(41.67)	107(32.92)	1.46(0.89–2.38)	0.135	
	Not adhered	49(58.33)	218(67.08)	1		

AOR: Adjusted Odds Ratio, CI: Confidence Interval.

#Others $Divorced and widowed.

*significant at P-value <0.05, COR: Crude Odds Ratio

**Table 3:** Factors associated with diabetic nephropathy in Ayder referral Hospital, Ethiopia, 2016.

**Variables**	**Category**	**Cases** **(n = 84)**	**Controls** **(n = 325)**	**COR (95% CI)**	**P- value**	**AOR (95% CI)**
**Adherence to blood glucose measurement**	Adhered	2(2.38)	40(12.31)	1	0.017	1
	Not adhered	82(97.62)	285(87.69)	5.75(1.36–21.32)		6.81(1.15–40.24)*
**Drink Alcohol**	Yes	49(58.33)	117(36.00)	2.49(1.53–4.06)	<0.001	
	No	35(41.67)	208(64.00)	1		
**Type of DM**	Type I	12(14.29)	124(38.15)	1		
	Type II	72(85.71)	201(61.85)	3.7(1.93–7.1)	<0.001	
**Duration of DM after Diagnosis**		84	325	1.13(1.077–1.18)		1.09(1.036–1.15)*
**Family history of DM**	Yes	11(13.10)	42(12.92)	1.02(0.5–2.07)	0.967	
	No	73(86.90)	283(87.08)	1		
**Other Complications**	Yes	52(61.90)	102(31.38)	3.55(2.16–5.85)	<0.001	
	No	32(38.10)	223(68.62)	1		
**Systolic Hypertension**	Yes	40(47.62)	80(24.62)	2.78(1.69–4.58)	<0.001	2.13(1.002–4.51)*
	No	44(52.38)	245(75.38)	1		1
**Diastolic Hypertension**	Yes	20(23.81)	52(16.00)	1.64(0.92–2.94)	0.096	0.45(0.19–1.06)
	No	64(76.19)	273(84.00)	1		1
**Glycemic control**	Poor	58(69.05)	163(50.15)	2.22(1.33–3.7)	0.002	2.71(1.49–4.95)*
	Good	26(30.95)	162(49.85)	1		1
**BMI**	Underweight	2(47.62)	45(13.85)	0.25(0.06–1.07	0.061	0.57(0.12–2.73)
	Normal	40(2.38)	224(68.92)	1		1
	Overweight	42(50.00)	56(17.23)	4.2(2.49–7.08)	<0.001	2.7(1.47–4.96)*

AOR: Adjusted Odds Ratio, CI: Confidence Interval.

*significant at P-value <0.05, COR: Crude Odds Ratio

The final multiple logistic regression model was fitted using the model Hosmer and Lemeshow test with a p-value of 0.52, Omnibus test p-value < 0.001 and the percentage of the model that was accurately classified was 83.4% that fulfils the criteria of accuracy. No detected multicollinearity. The maximum reported VIF was 3.045 and standard error of Beta coefficients of the model which has maximum standard error 1.24.

## Discussion

In this study, increasing in age and duration of DM after diagnosis, poor adherence to blood sugar measurement at home, having systolic hypertension, poor glycemic control and overweight determines the presence of nephropathy in diabetic patients.

Every one year increment in age of diabetic patient, development of diabetic nephropathy increased by 3.7%; that means for 10 years increase in duration of diabetes after diagnosis, the probability of developing diabetic nephropathy increases by 1.43 times (43%). Many studies conclude that older age is a risk for developing diabetic nephropathy. As age increases, patients will be at greater probability of developing kidney diseases which are mostly reflected by proteinuria. With the diabetes inter-playing, older age patents then will be at increased risk to develop nephropathy as also evidenced elsewhere [7,18,19]. Adherence to blood glucose measurement at home was also predictor for developing nephropathy. Those not adhered to blood glucose measurement at home were 5.75 times more likely in developing nephropathy than the adhered ones. Practically since the access of glucometer to patients is very low the adherence is very low in both cases and controls which gave wide confidence interval of odds ratio.

This study shows that as the duration after diagnosis in years increases the probability of developing nephropathy in diabetic patients. One year increases in duration of diabetes after diagnosis increases the development of diabetic nephropathy by 1.09 times (9%) that means for 10 years increase in duration of diabetes after diagnosis the probability of developing diabetic nephropathy increases2.43times.This study is similar with other studies that conclude duration of diabetes after diagnosis predicts the development of diabetic nephropathy[7,13,15,18,20,26].The presence of systolic hypertension increased the probability of developing diabetic nephropathy. This is similar with studies in India, Cameron and Ethiopia[18,20,26].

Glycemic control was the other variable that determines the development of diabetic nephropathy. Poor glycemic control had highest probability of developing nephropathy than those with good glycemic control. This finding is supported by studies in urban China, Pakistan, India and Africa[13,15,17–19,26].However glycemic control is not independent predictor of diabetic nephropathy on the retrospective study in Shakiso Health center. This difference is due to sample size, setting in which our study is in referral Hospital that is due to comparability difference of the source population [20].

Body mass index also the other determine the development of diabetic nephropathy in DM patients. The probability of developing diabetic nephropathy is higher in over weight than the normal weight but there is no significance difference in normal and underweight of patients. This finding is supported by the studies like, systematic review article in Africa[17,18]. But body mass index is not independent predictor on the retrospective study in Shakiso Health center. This difference is due to sample size, setting in which our study is in referral Hospital[20].

In other studies, type of diabetes mellitus has associations on the development of diabetic nephropathy. But in this study, it is not independent predictor of diabetic nephropathy. This difference may be introduced by sample size and source population of the studies. Many of the studies were cross-sectional studies and some of the studies focus on separate type of diabetes.

In conclusion, in the light of these findings, targeted interventions should be designed at the follow up clinic to address the risk of developing diabetic nephropathy among the risk groups.

## Supporting information

S1 Data The minimal anonymized data set used for analyses. (DTA)
